# Concomitant aorto-right subclavian artery bypass with off-pump coronary artery bypass grafting: a case report

**DOI:** 10.1186/s13019-017-0650-0

**Published:** 2017-10-11

**Authors:** Hirokazu Tazume, Ken Okamoto, Toshihiro Fukui

**Affiliations:** Department of Cardiovascular Surgery, Kumamoto University Hospital, 1-1-1 Honjo, Kumamoto, 860-8556 Japan

**Keywords:** Aorto-right subclavian artery bypass, Brachiocephalic artery stenosis, Off-pump coronary artery bypass grafting, Case report

## Abstract

**Background:**

Atherosclerotic stenosis of the brachiocephalic artery sometimes occurs in patients with coronary artery disease, and can cause stroke during the perioperative period of coronary artery bypass grafting.

**Case presentation:**

We describe the case of a 77-year old male with severe stenosis of the brachiocephalic artery and severe coronary artery disease. He successfully underwent aorto-right subclavian artery bypass that was performed concomitantly with off-pump coronary artery bypass.

**Conclusion:**

Concomitant aorto-subclavian artery bypass with off-pump coronary artery bypass grafting is a therapeutic option that minimizes the risk of perioperative stroke in patients with brachiocephalic artery stenosis and coronary artery disease.

## Background

Atherosclerotic stenosis of the brachiocephalic artery can be associated with coronary artery disease [1]. Stenosis of the brachiocephalic artery can be treated via endovascular intervention; however, open bypass surgery has been shown to be a safe and effective option, especially in patients with extensive multivessel involvement [2]. Herein, we report a case of a patient with brachiocephalic artery stenosis and multiple coronary artery stenosis who successfully underwent aorto-right subclavian artery bypass performed concomitantly with off-pump coronary artery bypass grafting (CABG).

## Case presentation

**Fig. 1:**
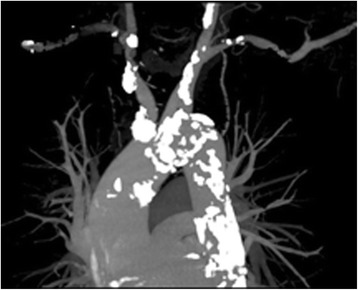
Preoperative computed tomography image showing severe calcification of the aortic arch and supra-aortic trunks

A 77-year old male was referred to our hospital for known coronary artery disease and a history of antero-septal myocardial infarction and syncope. Coronary angiography showed severe triple vessel disease. Computed tomography revealed severe diffuse calcification from the aortic arch to the abdominal aorta. In particular, just proximal brachiocephalic artery was shown to be circumferentially calcified and 90% stenosed (Fig. 1). Proximal left carotid artery and left subclavian artery were also shown to be calcified. But there were no significant stenosis at the origin of right carotid artery and vertebral artery. The peak blood pressure of the right arm was 40 mmHg lower than that of the left arm. Carotid ultrasonographic examination revealed 50% stenosis of right internal carotid artery and subclavian steal phenomenon in right vertebral artery. And post stenotic high flow pattern was observed in proximal brachiocephalic artery. We planned to perform an aorto-brachial or aorto-right subclavian artery bypass using a prosthetic graft and concomitant off-pump CABG.

A median sternotomy was performed, and the bilateral internal thoracic arteries were dissected. At the same time, a saphenous vein graft was dissected. A direct ultrasonographic examination revealed severe atheromatous plaques inside the brachiocephalic artery; hence, we chose the right subclavian artery as the distal anastomosis site for the prosthetic bypass conduit. An 8-mm polytetrafluoroethylene graft was anastomosed to the right subclavian artery, and then the proximal end of this graft was anastomosed to the aorta with a side-biting clamp. Off-pump CABG was then performed using a method described previously [3]. The left internal thoracic artery and the free right internal thoracic artery were anastomosed to the left anterior descending artery and the left circumflex artery, respectively. Two saphenous vein grafts were used for the diagonal branch and the distal branches of the right coronary artery. Proximal anastomoses between the saphenous vein grafts and the aorta were performed with proximal suturing devices. Lastly, a proximal anastomosis of the free right internal thoracic artery to the saphenous vein graft as a V-composite graft was performed [3].

**Fig. 2:**
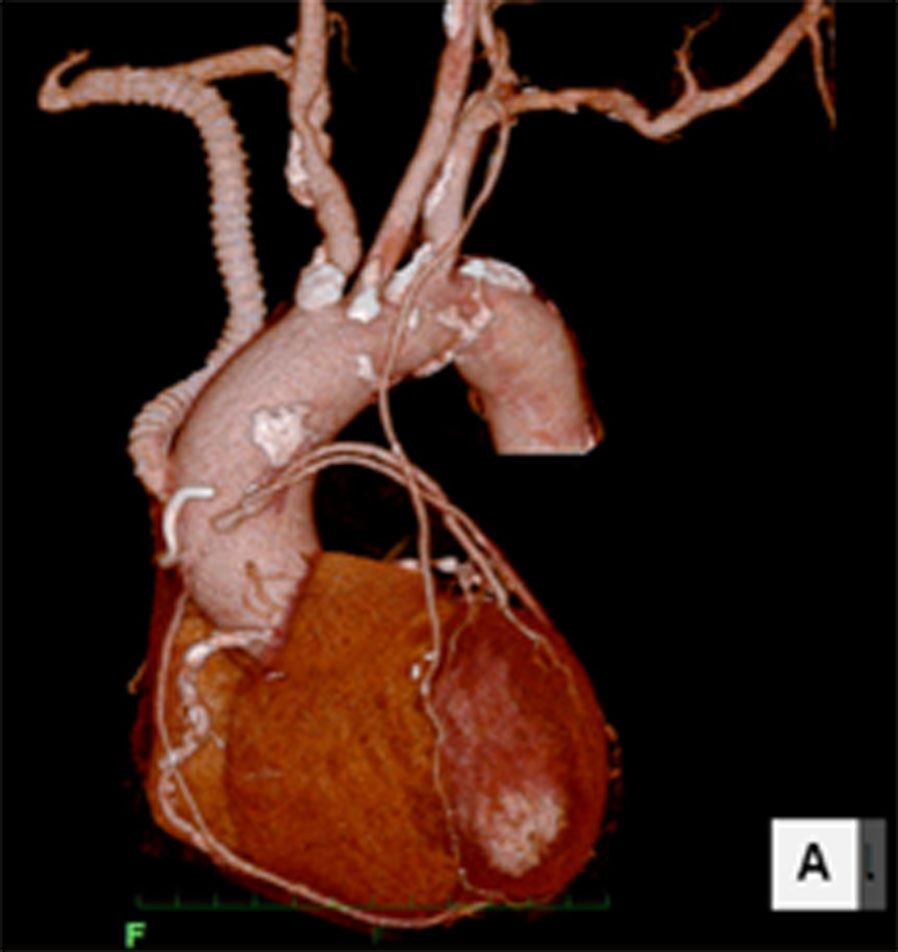
Postoperative computed tomography image showing patency of the aorto-right subclavian artery bypass and coronary artery bypass grafts

Postoperatively, no neurological deficit was observed. The blood pressures of the bilateral upper extremities were identical. Computed tomography demonstrated the patency of the coronary bypass grafts and the aorto-right subclavian artery bypass graft (Fig. 2). Postoperative radionuclide imaging showed no cerebral ischemia.

## Discussion

Atherosclerotic stenosis of the subclavian artery or brachiocephalic trunk is sometimes associated with coronary artery disease [3]. Many patients with stenosis of the subclavian artery or brachiocephalic trunk are asymptomatic; however, the stenosis can cause stroke during CABG. Several effective treatment methods have been established, including endovascular intervention, extra-anatomic bypass, or endarterectomy. In the present case, we performed aorto-right subclavian artery bypass concomitantly with off-pump CABG to minimize the risk of perioperative stroke. Modarai et al. [4] demonstrated that extra-anatomic bypass for supra-aortic trunk disease had a better patency and lower complication rate compared with percutaneous endovascular intervention. They showed that the patency rate of an extra-anatomic graft was 97% after a mean follow-up of 5 years [4]; however, their open surgical methods included only cervical approaches, such as carotid-subclavian and subclavian-subclavian artery bypass. Berguer et al. [2] reported that, in comparison to the cervical approach, the transthoracic approach has a relatively higher morbidity but more durable results in atherosclerotic disease involving complex and multiple supra-aortic trunk vessels. Khalil et al. [5] reported successful open surgery with a transthoracic approach in three patients using an aorto-subclavian or aorto-innominate artery bypass. Aorto-supra aortic trunk bypass is reportedly safe in patients with concomitant carotid disease with reduced cerebral risks, avoids endarterectomy and its attendant thrombotic risks, and provides a more physiological blood flow pattern than axillo-axillary or carotid-subclavian bypass [5]. In our patient, a direct ultrasonographic examination revealed severe atheromatous plaques inside the brachiocephalic trunk, and so we chose the right subclavian artery as the distal anastomosis site for the prosthetic bypass conduit. Moreover, ultrasound showed that the ascending aorta did not have any atheromatous plaques. Hence, we used the ascending aorta as the proximal anastomosis sites for the prosthetic graft and saphenous vein grafts.

Preoperative computed tomography revealed severe calcification just proximal brachiocephalic artery, and carotid ultra sound examination revealed subclavian steal phenomenon at right vertebral artery. But computed tomography revealed no significant stenosis from end of the brachiocephalic trunk to origin of right carotid artery and subclavian artery, and so we expected that the aorto-subclavian bypass provided a physiological brain perfusion and reduced the risk of stroke.

Takach et al. [6] reported the outcomes of patients with brachiocephalic and coronary artery disease who underwent concomitant brachiocephalic reconstruction and CABG. They demonstrated favorable early and mid-term outcomes of those patients; however, early mortality and stroke rate was 4.2% and 2.1%, respectively. They used cardiopulmonary bypass in all patients. Our strategy for isolated CABG is off-pump technique to minimize the postoperative stroke rate and other morbidities associated with cardiopulmonary bypass. However, hypotension during anastomosis is sometimes observed in off-pump procedure. In the present case, aorto-right subclavian artery bypass was performed before off-pump CABG to secure the cerebral blood flow. We believe that aorto-subclavian artery bypass with off-pump CABG is a therapeutic option that minimizes the risk of perioperative stroke in this patient.

## Conclusion

Concomitant aorto-subclavian artery bypass with off-pump CABG is a therapeutic option that minimizes the risk of perioperative stroke in patients with brachiocephalic artery stenosis and coronary artery disease.

## Abbreviations

CABG: Coronary artery bypass grafting
